# Mouse Oocyte Methylomes at Base Resolution Reveal Genome-Wide Accumulation of Non-CpG Methylation and Role of DNA Methyltransferases

**DOI:** 10.1371/journal.pgen.1003439

**Published:** 2013-04-18

**Authors:** Kenjiro Shirane, Hidehiro Toh, Hisato Kobayashi, Fumihito Miura, Hatsune Chiba, Takashi Ito, Tomohiro Kono, Hiroyuki Sasaki

**Affiliations:** 1 Division of Epigenomics, Medical Institute of Bioregulation, and Epigenome Network Research Center, Kyushu University, Fukuoka, Japan; 2 Graduate School of Medical Sciences, Kyushu University, Fukuoka, Japan; 3 Department of Bioscience, Tokyo University of Agriculture, Tokyo, Japan; 4 Department of Biophysics and Biochemistry, Graduate School of Science, The University of Tokyo, Tokyo, Japan; 5 Department of Informative Genetics, Environment and Genome Research Center, Tohoku University Graduate School of Medicine, Sendai, Japan; 6 Genome Research Center, NODAI Research Institute, Tokyo University of Agriculture, Tokyo, Japan; University of Pennsylvania, United States of America

## Abstract

DNA methylation is an epigenetic modification that plays a crucial role in normal mammalian development, retrotransposon silencing, and cellular reprogramming. Although methylation mainly occurs on the cytosine in a CG site, non-CG methylation is prevalent in pluripotent stem cells, brain, and oocytes. We previously identified non-CG methylation in several CG-rich regions in mouse germinal vesicle oocytes (GVOs), but the overall distribution of non-CG methylation and the enzymes responsible for this modification are unknown. Using amplification-free whole-genome bisulfite sequencing, which can be used with minute amounts of DNA, we constructed the base-resolution methylome maps of GVOs, non-growing oocytes (NGOs), and mutant GVOs lacking the DNA methyltransferase Dnmt1, Dnmt3a, Dnmt3b, or Dnmt3L. We found that nearly two-thirds of all methylcytosines occur in a non-CG context in GVOs. The distribution of non-CG methylation closely resembled that of CG methylation throughout the genome and showed clear enrichment in gene bodies. Compared to NGOs, GVOs were over four times more methylated at non-CG sites, indicating that non-CG methylation accumulates during oocyte growth. Lack of Dnmt3a or Dnmt3L resulted in a global reduction in both CG and non-CG methylation, showing that non-CG methylation depends on the Dnmt3a-Dnmt3L complex. Dnmt3b was dispensable. Of note, lack of Dnmt1 resulted in a slight decrease in CG methylation, suggesting that this maintenance enzyme plays a role in non-dividing oocytes. Dnmt1 may act on CG sites that remain hemimethylated in the *de novo* methylation process. Our results provide a basis for understanding the mechanisms and significance of non-CG methylation in mammalian oocytes.

## Author Summary

Methylation of cytosine bases in DNA is an epigenetic modification crucial for normal development, retrotransposon silencing, and cellular reprogramming. In mammals, the vast majority of 5-methylcytosine occurs at CG dinucleotides, and thus most studies to date have focused on this dinucleotide. However, recent studies have shown that 5-methylcytosine is abundant at non-CG (CA, CT, and CC) sites in certain tissues and certain cell types in human and mouse. We previously identified non-CG methylation in CG-rich sequences, including the imprint control regions in mouse germinal vesicle oocytes, but its global distribution and the enzymes responsible are unknown. Using advanced high-throughput sequencing technology applicable to minute amounts of DNA, we obtained high-resolution methylation maps of newborn non-growing oocytes, adult germinal vesicle oocytes, and mutant germinal vesicle oocytes lacking any of the four DNA methyltransferase family proteins. Our results revealed that non-CG methylation accumulates genome-wide in close proximity to highly methylated CG sites during the oocyte growth stage. We also found that the *de novo* DNA methyltransferase proteins Dnmt3a and Dnmt3L are responsible for non-CG methylation in oocytes. Unexpectedly, we found that the maintenance methyltransferase Dnmt1 has a role in *de novo* CG methylation. Our study provides a basis for understanding the mechanisms and significance of non-CG methylation in mammalian oocytes.

## Introduction

DNA methylation is a well-characterized epigenetic modification crucial for normal mammalian development, retrotransposon silencing, and cellular reprogramming [1], [2]. In mammals, a high proportion of 5-methylcytosines (mCs) occurs at CG dinucleotides, and thus studies on DNA methylation so far have focused on this dinucleotide. However, recent advances in the high -throughput DNA sequencing technology changed the scene [3], [4]. “Methylome” analyses using whole-genome bisulfite sequencing (WGBS) showed that mC occurs at non-CG sites, in addition to CG sites, in human and mouse embryonic stem (ES) cells, human induced pluripotent stem (iPS) cells, and mouse brain [5]–[9]. Moreover, we previously reported the prevalence of non-CG methylation in mouse germinal vesicle oocytes (GVOs), notably at maternally methylated imprint control regions (ICRs) and some CG-rich island regions (CGIs) [10], [11].

Methylation of cytosine bases in CG dinucleotides is catalyzed by enzymes called DNA methyltransferases (Dnmts). Among these enzymes, Dnmt1 is the maintenance methyltransferase that copies the pre-existing methylation patterns upon DNA replication, while Dnmt3a and Dnmt3b are the *de novo* methyltransferases that create new methylation patterns. Another member of the family, Dnmt3L, lacks enzymatic activity, but enhances the activity of Dnmt3a and Dnmt3b [12], [13]. It is unknown which Dnmt is responsible for non-CG methylation in oocytes. Using the reduced representation bisulfite sequencing (RRBS) method, which focuses mainly on CG-rich regions, Smallwood *et al.* (2011) demonstrated that the *de novo* methylation at CG sites occurs in many CGIs during oocyte growth, and depends on Dnmt3a and Dnmt3L [14]. Using WGBS, we have shown that global CG methylation in GVOs appears to be Dnmt3L-dependent [11]. However, the genome-wide distribution pattern of non-CG methylation and the enzymes responsible for this methylation in oocytes remain unknown.

To answer these questions, we have used WGBS to construct the methylome maps in non-growing oocyte (NGOs), GVOs, and mutant GVOs lacking either Dnmt1, Dnmt3a, Dnmt3b, or Dnmt3L. We demonstrate that non-CG methylation occurs concomitantly with CG methylation during oocyte growth and that Dnmt3a and Dnmt3L are responsible for non-CG methylation. Our study also reveals a new function of Dnmt1 in *de novo* CG methylation.

## Results

### Amplification-free whole-genome bisulfite sequencing

To obtain methylome maps at single-base resolution from a limited number of oocytes, we used the post-bisulfite adaptor tagging (PBAT) method that requires only nanogram quantities of DNA for amplification-free WGBS [15]. The PBAT method was previously applied to four hundred GVOs and 19.3 million uniquely mapped reads were achieved [11]. To elucidate the developmental timing of non-CG methylation, we determined the methylome of newborn NGOs in addition to adult GVOs. Furthermore, to examine the role of the Dnmts in non-CG methylation, we determined the methylomes of *Dnmt1*-knockout GVOs (designated Dnmt1-KO), *Dnmt3a*-knockout GVOs (Dnmt3a-KO), *Dnmt3b*-knockout GVOs (Dnmt3b-KO), and *Dnmt3L*-knockout GVOs (Dnmt3L-KO). Using PBAT, we obtained 158–460 million uniquely mapped reads for the respective methylome (Table 1). The average read depths were 2.8×–8.3× per strand (Table 1). We were able to determine the methylation status of >77% of the genomic CG sites and >74% of the non-CG sites by at least one read (Table 1 and Figure S1).

**Table 1:** Sequencing and mapping summary.

Sample	Sequenced reads	Uniquely mapped reads	Average depth per strand	Covered CG per strand (at least 1 read) (%)	Covered non-CG per strand (at least 1 read) (%)	Conversion rate (%)
GVO	700,924,625	375,655,439	6.8	86.1	84.7	99.55
Dnmt1-KO	869,379,030	459,730,977	8.3	87.2	86.1	99.51
Dnmt3a-KO	413,607,279	215,309,614	3.9	80.2	78.0	99.54
Dnmt3b-KO	744,361,742	398,894,906	7.2	86.3	85.0	99.53
Dnmt3L-KO	350,109,037	186,146,358	3.3	78.8	76.1	99.57
NGO	343,554,795	158,167,157	2.8	77.1	74.2	99.13

To study non-CG methylation, a high rate of bisulfite conversion is essential because unconverted cytosines are counted as mCs. We therefore spiked each sample with unmethylated lambda phage DNA before the bisulfite reaction and calculated the conversion rate using this substrate. We confirmed that the conversion rate always exceeded 99% and, in most of the cases, 99.5% (Table 1). Judging from the data of the previous WGBS studies [5], [11], our conversion rates are sufficient to analyze non-CG methylation.

To assess the quality of our data further, we compared the data from this study with our previous locus-specific observations [10], [16]. Consistent with the previous data, the CG sites at the maternally methylated ICRs were hypermethylated (90–98%), whereas those at the paternally methylated ICRs were hypomethylated (1–5%) in GVOs (Table S1). In Dnmt3a-KO and Dnmt3L-KO samples, the maternally methylated ICRs were methylated at a rate of less than 20% (Table S1). In addition, CGIs in intragenic (gene body) regions were more methylated than those in intergenic or promoter regions, as previously reported [14] (Figure S2).

### Landscape of non-CG methylation in GVOs

**Figure 1:**
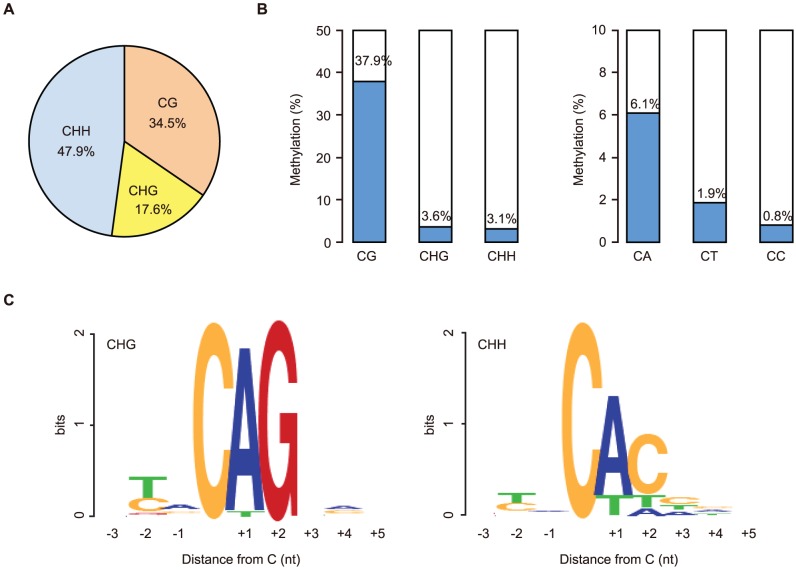
Abundant non-CG methylation in GVOs. (A) Proportions of mCs in contexts of CG, CHG, and CHH. Data for mCs at positions covered at least 4× on the same strand were used, and those with more than 100× coverage were excluded. (B) Levels of methylation at CG and non-CG sites. Non-CG sites were further divided into different tri- (CHG and CHH) and di-nucleotide sequences (CA, CT, and CC). (C) Bases neighboring the highly methylated (≥30%) non-CG sites.

We first focused on the methylome of GVOs for more detailed studies. Here 278 million (redundant) mCs were obtained in all mapped reads, of which 65.5% occurred at non-CG sites (17.6% at CHG and 47.9% at CHH; H = A, T, or C; Figure 1A). The average methylation level was 37.9% at CG, 3.6% at CHG, and 3.1% at CHH (Figure 1B). Among the non-CG sites, CA sites were methylated most often (6.1%), whereas CT and CC sites were less frequently methylated (1.9% and 0.8%, respectively); this was also a feature in human ES cells and mouse brain [5], [6], [9] (Figure 1B). When we examined the sequences around the methylated CHG/CHH sites, TACAGC and TACACC were the most frequently methylated (21.1% and 30.7%, respectively; Figure 1C).

**Figure 2:**
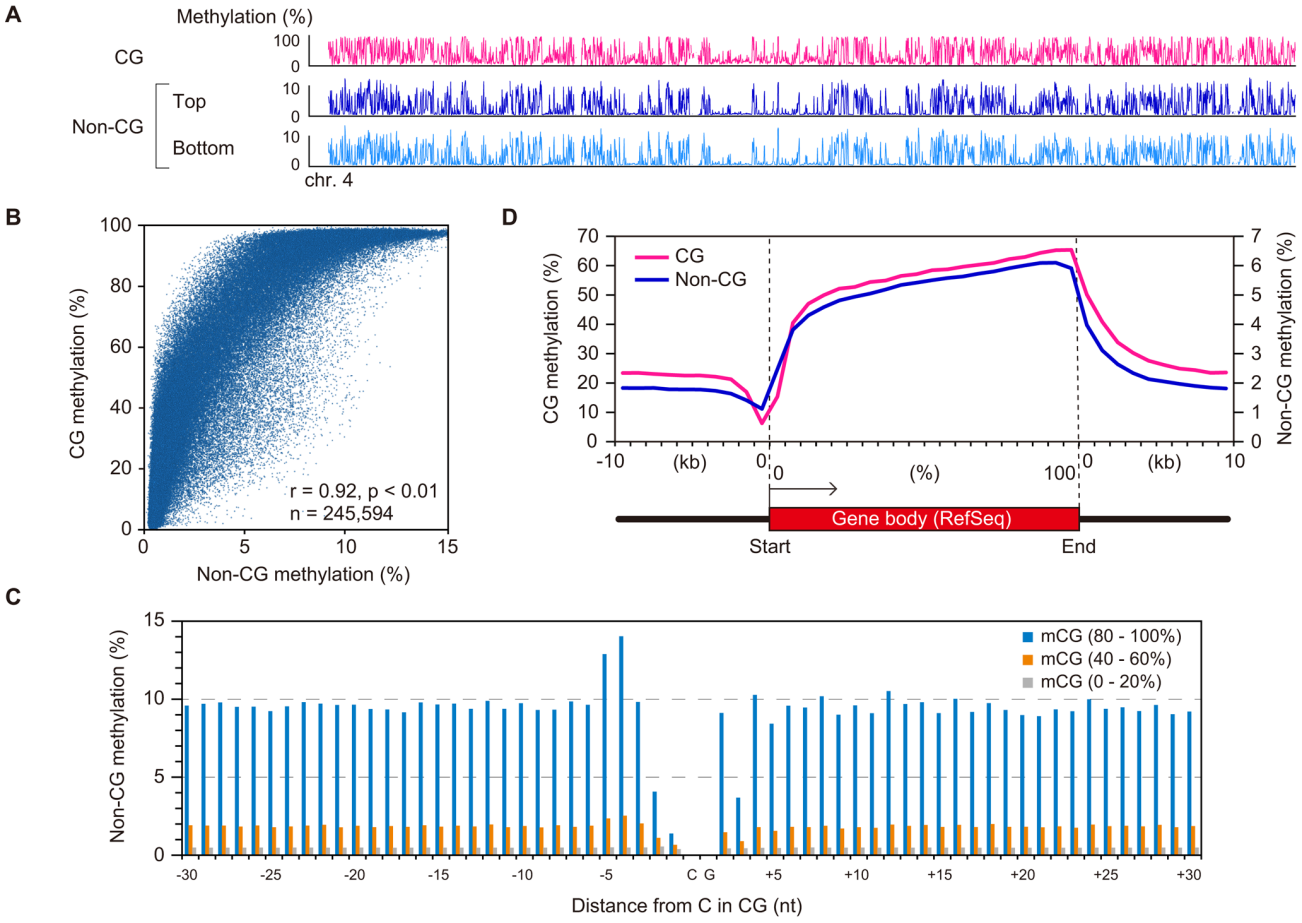
Relationship between CG methylation and non-CG methylation in GVOs. (A) Levels of CG methylation and non-CG methylation across the entire chromosome 4 in a non-overlapping sliding window of 50 kb. The two strands were separately analyzed for non-CG methylation. (B) Correlation between the levels of CG methylation and non-CG methylation in 10-kb non-overlapping sliding windows across the genome was indicated using Spearman's rank correlation coefficient. (C) Effect of CG on non-CG methylation at positions immediately upstream and downstream of mC sites. Blue, orange, and gray bars indicate the levels of non-CG methylation around CG sites with at least 10× coverage with methylation levels of 80–100%, 40–60%, and 0–20%, respectively. (D) Levels of CG methylation and non-CG methylation relative to gene structure. The upstream and downstream regions (10-kb each) were split into 10 non-overlapping windows to determine the methylation levels. The intragenic or coding regions were divided into 20 small windows for methylation analysis.

A chromosomal view of CG methylation and non-CG methylation in GVOs revealed large variations in methylation levels throughout entire chromosomes, and a concordance between CG and non-CG methylation at a 50-kilobase (kb) resolution (Figure 2A and Figure S3). Using sliding, non-overlapping windows of 10 kb along all chromosomes, we found a genome-wide correlation between CG methylation and non-CG methylation (Figure 2B). This is consistent with the previous reports that non-CG methylation is generally found in regions containing CG methylation in mouse ES cells [8], [17]. The concordance between CG methylation and non-CG methylation was also observed in CGIs (Figure S2). We then analyzed the effect of the level of methylation at a CG site on the levels of methylation at nearby non-CG sites. The non-CG methylation levels near the highly (>80%) and weakly (<20%) methylated CG sites were 9.3% and 0.5%, respectively (Figure 2C). Taken together, these results indicate that non-CG methylation is highly linked to CG methylation in GVOs. Interestingly, we found that the levels of non-CG methylation at positions −1, −2, and +3 of a CG site are low and those at positions −4 and −5 are high (Figure 2C). The extremely low level of methylation at position −1 is consistent with the low level of methylation of the first cytosine at CCG sites (Figure 1C), but we do not have plausible explanations for the other peaks or dips. We also determined the distribution of CG and non-CG methylation across the RefSeq genes and found that their methylation levels are lowest in the region immediately upstream of the transcription start site (TSS), and gradually increase towards the end of the last exon (Figure 2D). Thereafter, the levels of methylation drop markedly in the downstream region (Figure 2D). The actual level of non-CG methylation was 5.2% in the intragenic region (gene body) and 1.9% in the intergenic region, which is consistent with previous reports [5], [6].

**Figure 3:**
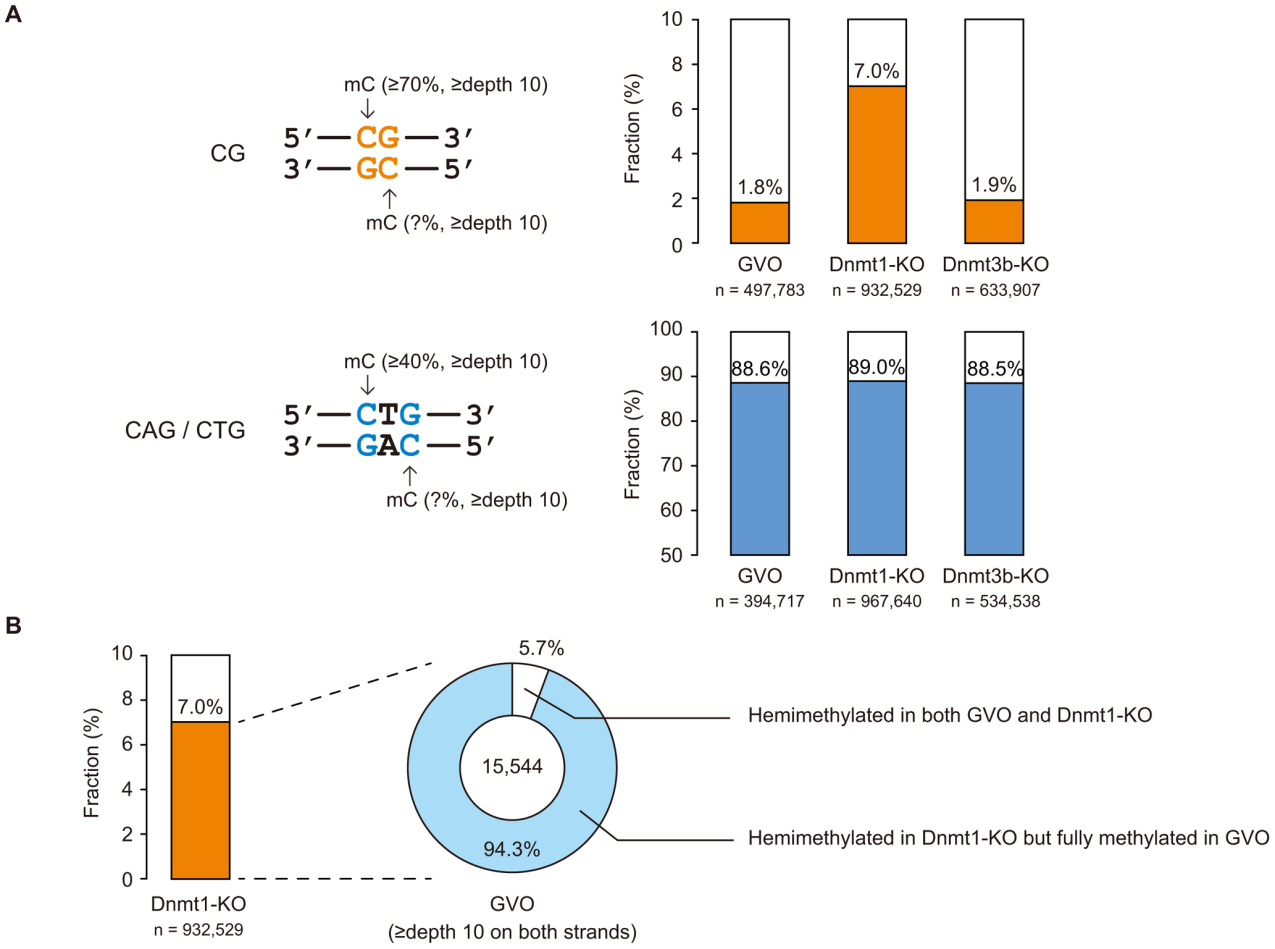
Strand asymmetry in methylation at CG and non-CG sites. (A) Fractions of CG and CAG/CTG sites showing strand asymmetry in methylation (hemimethylation) in GVOs, Dnmt1-KO, and Dnmt3b-KO. The data were taken from CG and CAG/CTG sites with methylation levels of ≥70% and ≥40%, respectively, on at least one strand, with a read depth of at least 10× for both strands (left). Bar charts show the hemimethylated fractions (right). Statistical differences in methylation between the strands were calculated using Fisher's exact test (p<0.05). Note that most CAG/CTG sites are hemimethylated in all samples and that CG sites show increased hemimethylation in Dnmt1-KO. (B) CG sites hemimethylated in Dnmt1-KO but not in GVOs. Dnmt1-KO demonstrated an increase in hemimethylated CG sites (orange, left), most of which were fully methylated in GVOs.

CHG sequences show partial strand-symmetry and have cytosines on both strands. We determined the methylation levels at CAG/CTG sites on the respective strands. We found that, while 98% of highly methylated CG sites (methylation level ≥70%) are methylated on both strands (Figure 3A), 89% of highly methylated CAG/CTG sites (methylation level ≥40% on one of the strands) are methylated only on one strand (Figure 3A), as previously reported in human ES cells [5].

### Non-CG methylation occurs during oocyte growth

**Figure 4:**
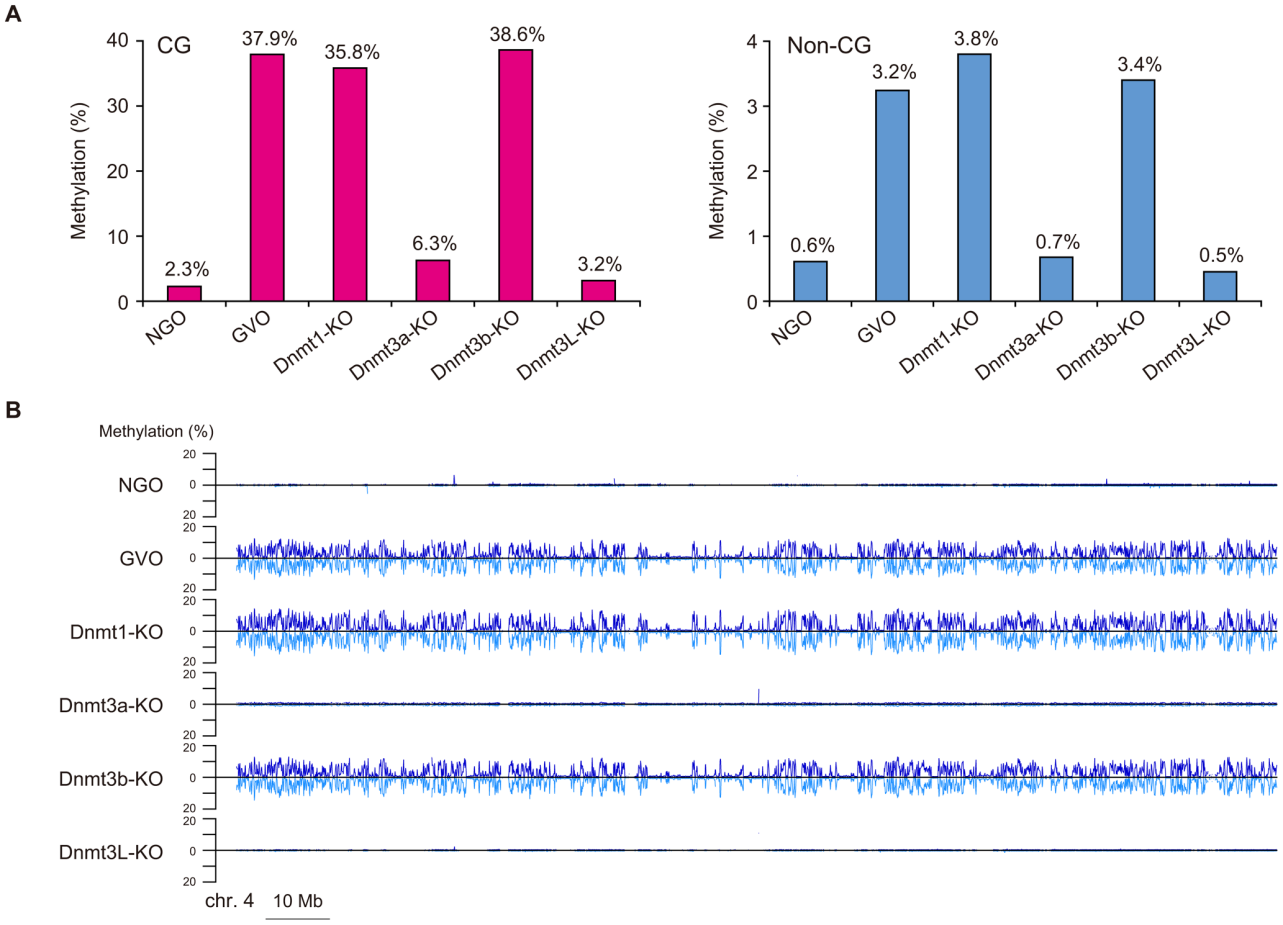
Comparison of the methylomes in NGOs, GVOs, and Dnmt-KO. (A) Overall levels of CG methylation and non-CG methylation in NGOs, GVOs, Dnmt1-KO, Dnmt3a-KO, Dnmt3b-KO, and Dnmt3L-KO. The data were not corrected for the bisulfite non-conversion rate (0.43–0.87, see Table 1). Thus, NGOs, Dnmt3a-KO, and Dmt3L-KO may actually have no or very little non-CG methylation. (B) Profiles of non-CG methylation along chromosome 4 determined using a non-overlapping sliding window of 50 kb in all samples. The data for the two strands are shown separately above and below the horizontal line.

It is known that the maternally methylated ICRs and a thousand non-imprinted CGIs acquire CG methylation during oocyte growth [14], [18]. We therefore determined the methylome of newborn NGOs (before growth) and compared it with that of GVOs (after growth). The level of CG methylation was 2.3% in NGOs, which increased to 37.9% in GVOs (Figure 4A; Also see Figure 1B), supporting the previous finding on CGIs [14]. Similarly, the level of non-CG methylation increased from 0.61% (NGOs) to 3.2% (GVOs; Figure 4A). (Note that the level is actually not above the non-conversion rate in NGOs (Table 1), suggesting that NGOs may actually have no non-CG methylation.) Thus, CG methylation and non-CG methylation occur at the same time during oocyte growth. The increase in non-CG methylation during this stage occurred globally (Figure 4B and Figure S4).

**Figure 5:**
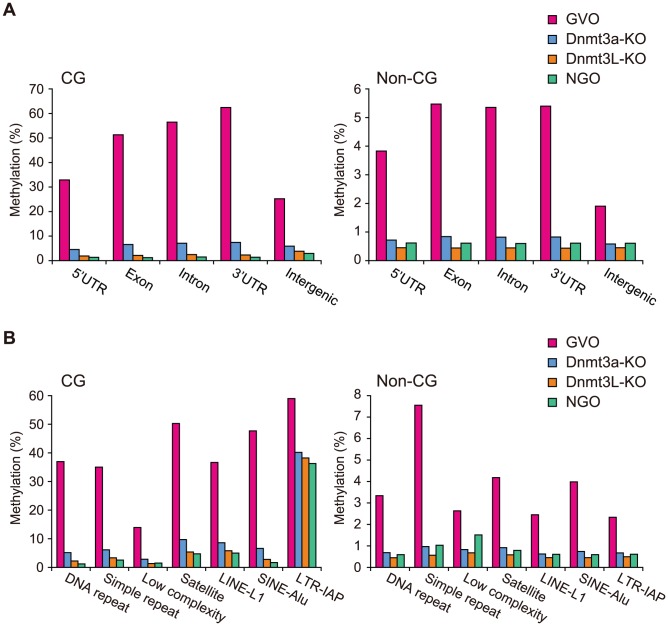
CG and non-CG methylation levels in different genomic elements in NGOs, GVOs, Dnmt3a-KO, and Dnmt3L-KO. (A) Levels of CG methylation and non-CG methylation in intragenic and intergenic regions. Intragenic regions are further divided into 5′UTRs, exons, introns, and 3′UTRs according to the RefSeq annotations. (B) Levels of CG methylation and non-CG methylation in different types of repetitive elements. Annotation for repetitive elements was obtained from the UCSC Genome Browser.

Next, we investigated the methylation changes during oocyte growth at different genomic elements. Both intragenic and intergenic regions demonstrated low levels of CG and non-CG methylation in NGOs, but higher levels in GVOs (Figure 5A). Most repetitive elements showed similar developmental changes (Figure 5B). However, intracisternal A particle (IAP) elements retained relatively high levels of CG methylation (36%) in NGOs, which indicates that approximately 62% of CG methylation found in IAPs in GVOs already exist in NGOs. This is consistent with the previous findings that IAPs are substantially resistant to epigenetic reprogramming in primordial germ cells [19]–[22]. Interestingly, even IAPs did not retain non-CG methylation in NGOs (Figure 5B). The maternally methylated ICRs generally showed low levels of CG methylation in NGOs, but the *Peg10* and *Impact* ICRs retained relatively high CG methylation (17–19%; Table S1). Both of these ICRs contain tandem repeats [23], [24], and these repeats had higher CG methylation levels (34% and 23%, respectively) than the rest of the ICRs in NGOs (Figure S5), although other ICRs such as *Igf2r*, *Kcnq1ot1*, and *Snrpn* also contain tandem repeats. Again, non-CG methylation was not high even at the *Peg10* and *Impact* ICRs in NGOs (Table S1). In general, there was no correlation between CG methylation and non-CG methylation in NGOs (Figure S6), which differs from the finding in GVOs (Figure 2B). Taken together, these results indicate that non-CG methylation is virtually absent in NGOs and accumulates during oocyte growth.

### Role of DNA methyltransferases in non-CG methylation

We next compared the methylomes of wild-type and mutant GVOs lacking Dnmts. Dnmt3a-KO and Dnmt3L-KO demonstrated a global reduction in both CG methylation and non-CG methylation, whereas Dnmt3b-KO showed no significant change (Figure 4A and Figure S4). In Dnmt3a-KO and Dnmt3L-KO, intragenic regions, intergenic regions, and repetitive elements (except IAP) all showed very low levels of methylation (Figure 5). Dnmt3a and Dnmt3L form a tetramer complex (composed of two molecules of each) and thereby act as an effective *de novo* CG methyltransferase [25]. Our results revealed that, not only CG methylation, but also non-CG methylation depends on this complex. Since non-CG methylation occurs on one strand, even at symmetrical CHG sites (Figure 3A), no maintenance methylation activity seems to exist. Thus, non-CG methylation is solely attributed to *de novo* methylation by the Dnmt3a-Dnmt3L complex.

Next, we separately identified and compared regions that showed significantly lower CG methylation and non-CG methylation in NGOs, Dnmt3a-KO, and Dnmt3L-KO, respectively, compared to GVOs, using a non-overlapping sliding window of 10 kb. We found that 80% (CG) and 92% (non-CG) of the regions identified in NGOs overlapped with the regions identified in both Dnmt3a-KO and Dnmt3L-KO, confirming that these regions are targeted by the Dnmt3a-Dnmt3L complex for *de novo* CG and non-CG methylation during oocyte growth (Figure S7).

Dnmt1 preferentially methylates hemimethylated CG sites and thus copies the pre-existing methylation patterns upon DNA replication. Interestingly, Dnmt1-KO showed a slightly lower level of CG methylation compared to GVOs (Figure 4A). This was associated with an increase in hemimethylated CG sites because the proportion of CG sites methylated on only one strand increased (Figure 3A). Most of the CG sites that were highly methylated on one strand in Dnmt1-KO were highly methylated on both strands in GVOs (Figure 3B). The findings were validated by conventional bisulfite sequencing of two selected loci (Figure S8). Because these hemimethylated CG sites were unmethylated in NGOs (Figure S8), the increase in hemimethylation is attributed to incomplete *de novo* methylation during oocyte growth. These results suggest that Dnmt1 acts on CG sites that have remained hemimethylated during the process of *de novo* methylation. Unexpectedly, Dnmt1-KO showed a slight increase in non-CG methylation (Figure 4A). This could be attributed to a secondary effect caused by compensatory up-regulation of Dnmt3a [26] (Figure S9).

## Discussion

Non-CG methylation in mouse oocytes was first identified in a few genes [27], [28], and then in several maternally methylated ICRs and two CGIs [10]. More recently, we have reported the abundant presence of non-CG methylation in mouse GVOs (3.4–3.8% methylation levels), which was revealed by WGBS using the PBAT method [11]. However, the detail of the genomic distribution, developmental timing, and enzymatic basis of non-CG methylation has been lacking.

To further investigate these facets of non-CG methylation, we have constructed and compared the methylome maps of GVOs, NGOs, and GVOs defective for either of the Dnmts (Dnmt1-KO, Dnmt3a-KO, Dnmt3b-KO, and Dnmt3L-KO). We found that a surprisingly high proportion (66%) of mCs occurs at non-CG sites in GVOs. This proportion is higher than that reported for human ES cells or mouse brain (ca. 30%) [5], [9]. When we examined the overall distribution of non-CG methylation, we found that non-CG methylation occurs almost exclusively in regions rich in CG methylation. This appears to be consistent with the fact that the same enzyme complex is responsible for both *de novo* CG and non-CG methylation in GVOs (see later). Furthermore, a comparison of the methylomes of NGOs and GVOs revealed that non-CG methylation occurs during the oocyte growth stage, concomitant with *de novo* CG methylation. This stage corresponds to meiotic prophase I, a stage in which oocytes remain non-replicating for an extended period (up to a year in mice).

Although CHG sites are more frequently methylated than CHH sites in human ES cells [5], we did not observe this trend in GVOs, which was consistent with the finding in mouse brain [9]. We found that the preferred sequence for non-CG methylation in GVOs is CA, and TACA(G/C)C in particular. This was also true in human ES cells and mouse brain [9], [29], [30], and thus the motif may be the common site for non-CG methylation in many mammalian species. Interestingly, the preference for thymine at position −2 and cytosine at position +3 is reminiscent of the sequence preference of Dnmt3a [31]; furthermore, murine Dnmt3a has been shown to mediate non-CG methylation in ES cells [32]. Indeed, we found Dnmt3a and its regulatory protein Dnmt3L to be responsible for non-CG methylation in GVOs. This was consistent with the role of the Dnmt3a-Dnmt3L complex in *de novo* CG methylation during oocyte growth [11], [14], [16]. Dnmt3b was dispensable, perhaps because of the extremely low expression of this gene in oocytes [33]. Consistent with this, the *Dnmt3b* promoter (±2 kb of TSS) showed a high level of CG methylation (>93%), whereas other *Dnmt* promoters showed lower methylation (<29%; data not shown). This is in contrast to the recent reports that non-CG methylation depends on both Dnmt3a and Dnmt3b in human ES cells [30] and on Dnmt3a, Dnmt3b, and Dnmt3L in mouse ES cells [17].

Based on the crystallographic analysis of the Dnmt3a-Dnmt3L complex, a model was proposed in which the two active sites of the complex recognize and methylate two CG sites spaced 8–10 base pairs apart [25]. Although Dnmt3a and Dnmt3L act in the same complex, the CG and non-CG methylation levels in Dnmt3a-KO were higher than those in Dnmt3L-KO. This could be explained by the difference in knockout strategy, as conventional knockout GVOs (Dnmt3L-KO) may show a more severe phenotype than conditional knockout GVOs (Dnmt3a-KO), depending on the efficiency of the Cre-mediated deletion in the conditional knockout.

Interestingly, the level of CG methylation was slightly lower in Dnmt1-KO than GVOs, and this was correlated with an increase in hemimethylated sites. We found that CG sites located in intergenic regions or retrotransposons are more likely to be hemimethylated than those in gene bodies in Dnmt1-KO (data not shown). It seems that Dnmt1 methylates CG sites that have remained hemimethylated during the *de novo* methylation process in oocyte. Uhrf1 (Np95) recognizes hemimethylated CG sites and helps to recruit Dnmt1 in somatic cells [34], [35]. Since Uhrf1 is also expressed in GVOs [11], [36], Dnmt1 could utilize this protein to recognize hemimethylated CG sites in GVOs. Thus, Dnmt1 has a role, even in a non-replicating (and non-dividing) cell type, in establishing fully methylated CG sites. Unexpectedly, the level of non-CG methylation was slightly higher in Dnmt1-KO compared to GVOs. Because a loss of Dnmt1 can cause up-regulation of Dnmt3a [26] (Figure S9), this gain in non-CG methylation could be attributed to this compensation mechanism.

Based on the data from this and other studies, we speculate that there are two important prerequisites for the presence of non-CG methylation in mammalian cells. The first requirement is a high level of expression of Dnmt3a and/or Dnmt3b, whose target sequence specificity is not strict [37], [38]. All mammalian cells that have non-CG methylation meet this criterion. The second is that the cells should be non-replicating or slowly replicating. Since non-CG methylation occurs only on one strand, even at symmetric CHG sites, there appears to be no maintenance mechanism for non-CG methylation. Thus, non-CG methylation needs to be re-established *de novo* after each cell division. Among the cells that contain non-CG methylation, oocytes and neurons are post-replicative and do not divide. Human pluripotent cells replicate more slowly than mouse pluripotent cells [39], and thus have higher levels of non-CG methylation. Presumably, the level of non-CG methylation is determined by the balance between the activity of Dnmt3a/Dnmt3b and the rate of cell division, and this may be the reason why mouse pluripotent cells, which divide faster, have lower levels of non-CG methylation. Consistent with this, non-CG methylation in oocytes is lost in a replication dependent way during the cleavage stage [10], [40]. This is similar to what we recently observed in mouse testis, where non-CG methylation accumulates in non-dividing germ cells but becomes reduced after the resumption of mitosis [41].

Lastly, 5-hydroxymethylcytosine (hmC) has been identified as an important intermediate for passive, and potentially active, demethylation [42]. Since bisulfite sequencing cannot distinguish between mC and hmC, we could not determine whether hmC is present in oocytes. Fine mapping of hmC at CG and non-CG sites in oocytes will require development of new technologies because currently available methods are not adapted for limited amounts of DNA [43].

In summary, we have determined the methylome maps of mouse NGOs and GVOs and revealed the genome-wide distribution and developmental timing of non-CG methylation. Using GVOs lacking either of the Dnmt proteins, we identified Dnmt3a and Dnmt3L as the proteins responsible for non-CG methylation during oocyte growth. At this point in time, it is unclear whether non-CG methylation is a by-product of CG methylation or has any biological role. Our data will provide a basis for understanding the mechanism and role of non-CG methylation in mammals.

## Materials and Methods

### Oocytes

NGOs and GVOs were collected from 0–3-day old and over 8-week old C57BL/6 females (Clea, Japan), respectively. Mutant GVOs, designated Dnmt1-KO, Dnmt3a-KO, Dnmt3b-KO, and Dnmt3L-KO, were obtained from [*Dnmt1^2lox/2lox^, Zp3-Cre*] females, [*Dnmt3a^2lox/2lox^, Zp3-Cre*] females, [*Dnmt3b^2lox/2lox^, Zp3-Cre*] females, and *Dnmt3L*-null homozygous females, respectively [16], [33], [44]. The reason for the use of conditional knockout mice for *Dnmt1*, *Dnmt3a*, and *Dnmt3b* was the lethality of the relevant conventional knockout mice [16], [45]–[47]. In contrast, the conventional knockout mice for *Dnmt3L* are viable [44], [48].

### Preparation of the PBAT library

Libraries for WGBS were prepared using the PBAT method, as described previously [15]. Approximately one-thousand oocytes were spiked with 1 ng of unmethylated lambda phage DNA (Promega), placed in a lysis solution (0.1% SDS, 1 mg/mL proteinase K, 1 µg tRNA) and incubated for 60 min at 37°C, and then 15 min at 98°C, followed by bisulfite treatment using MethylCode Bisulfite Conversion Kit (Invitrogen). Bisulfite-treated DNA was double-stranded with Klenow fragment [3′→5′ exo(-); New England Biolabs], using BioPEA2N4 (5′-biotin-ACACTCTTTCCCTACACGACGCTCTTCCGATCTNNNN-3′) (first strand). The biotinylated strand was captured using Dynabeads M-280 Streptavidin (Invitrogen) and double-stranded with Klenow fragment (3′→5′ exo(-)), with PE-reverse-N4 (5′-CAAGCAGAAGACGGCATACGAGATNNNN-3′) (second strand). After removing the first strand, the second strand was used as a template for primer extension by Phusion Hot Start High-Fidelity DNA Polymerase (Finnzymes) with Primer-3 (5′-AATGATACGGCGACCACCGAGATCTACACTCTTTCCCTACACGACGCTCTTCCGATCT-3′). Concentrations of the PBAT libraries were measured by quantitative PCR (qPCR) using PE-forward and PE-reverse primers (Illumina) [15]. The PhiX v2 Control Kit (Illumina) was used as a standard for quantification.

### High-throughput sequencing and methylation analysis

All sequencing runs were single-ended and 100 nucleotides (nt) in length, and performed on the Illumina HiSeq 2000 platform. Based on the qPCR quantification, 3×10^8^ copies of double-stranded DNA from the PBAT library were sequenced per lane on HiSeq 2000, as previously described [15]. Cluster generation and sequencing were performed in single-read mode using the TruSeq SR Cluster Kit v3-cBot-HS (Illumina) and the TruSeq SBS Kit v3-HS (Illumina) according to the manufacturer's protocols. Sequenced reads were processed using the standard Illumina base caller (v.1.8.2). We truncated raw reads to 92 nt to remove lower quality bases near the end of the reads and any remaining adapter sequences incorporated in the read. The resulting reads were aligned to the reference genome (mouse mm9) using Bismark alignment software v.0.6.3 [49] with a maximum of two mismatches, and only uniquely aligned reads were retained. We estimated bisulfite conversion rates using reads that uniquely aligned to the lambda phage genome. For strand-independent analysis of CG methylation, counts from the two cytosines in a CG and its reverse complement were combined. We subsequently evaluated only CG sites with at least 6× coverage and non-CG sites with at least 4× coverage, and discarded cytosines with more than 100× coverage. CG islands, RefSeq genes, and repeat sequences for the mm9 genome were downloaded from the UCSC Genome Browser [50]. The logo plot images were generated with WebLogo [51].

### Accession number

Sequence data in this study have been deposited in DDBJ/GenBank/EMBL under accession number DRA000570.

## Supporting Information

Figure S1 Coverage and read counts of CG and non-CG sites in each sample. The percentages of cytosines in CG and non-CG sites that are covered by differing minimum number of reads (x axis) are shown by orange and blue bars, respectively.
(EPS)

Figure S2 Levels of CG and non-CG methylation in CGIs at different genomic contexts in GVOs. All CGIs were grouped into three classes according to the genomic contexts (promoter, intragenic, and intergenic). Each CGI class was then examined for the inclusion of CGIs with different levels of methylation. A promoter CGI contains an annotated TSS in the RefSeq or overlaps with a 2-kb region immediately upstream of an annotated TSS. An intragenic CGI overlaps with an annotated gene but does not contain its TSS. The remaining CGIs are defined as intergenic CGIs.
(EPS)

Figure S3 CG and non-CG methylation profiles along all chromosomes in GVOs. Levels of CG methylation (red) and non-CG methylation (top strand only; blue), determined in 50-kb windows, are plotted along all chromosomes.
(EPS)

Figure S4 Circular representation of CG methylation and non-CG methylation across all chromosomes. The level of methylation in 50-kb windows along all chromosomes is shown. The circles represent methylation at CG (outermost circle), non-CG (top strand) (second circle), and non-CG sites (bottom strand; innermost circle). The chromosome numbers are indicated.
(EPS)

Figure S5 CG methylation profiles of the *Impact* and *Peg10* ICRs in NGOs. Levels of CG methylation at the *Impact* and *Peg10* ICRs in NGOs are shown. Methylated and unmethylated CG sites are shown in filled and open circles, respectively. Red lines show the CG methylation values calculated in a sliding window of 300 nt and a step size of 50 nt along the ICRs. Orange, blue, and green broad arrows represent tandem repeats, simple repeats, and low complexity sequences, respectively. Note that CG methylation is retained within and around the tandem repeats. No IAP element or IAP-like structure was found in the ICRs.
(EPS)

Figure S6 Genome-wide correlation between CG and non-CG methylation levels. Level of non-CG methylation was plotted against the level of CG methylation in every 10-kb window to indicate correlation (Spearman's rank correlation coefficient).
(EPS)

Figure S7 Identification and comparison of regions less methylated in NGOs, Dnmt3a-KO, and Dnmt3L-KO, compared to GVOs. Venn diagrams indicate the overlaps between the regions less methylated in NGOs, Dnmt3a-KO, and Dnmt3L-KO, compared to GVOs. The results are shown separately for CG methylation and non-CG methylation. Non-overlapping 10-kb sliding windows along all chromosomes were analyzed. Fisher's exact test (p<0.01) was used to determine the statistical significance of differences in methylation.
(EPS)

Figure S8 Bisulfite sequencing of the top and bottom strands at two selected loci. Conventional bisulfite sequencing of the top and bottom strands at the *Igf1r* (A) and *Atp8b1* (B) introns in GVOs, Dnmt1-KO, and NGOs. Methylated and unmethylated CG sites are shown in filled and open circles, respectively. Three to five hundred oocytes were lysed and directly bisulfite converted. Equivalent of 30–50 oocytes was used as a template for PCR consisted of 8 cycles of 95°C for 30 sec, 60–56°C (with 0.5°C decrement per cycle) for 30 sec, and 72°C for 30 sec, followed by 32 cycles of 95°C for 30 sec, 56°C for 30 sec, and 72°C for 30 sec. The PCR products were cloned into pMD20 (Takara) and sequenced. The quality of each sample was verified by the methylation levels at the *Igf2r* and *H19* ICRs, which should show nearly 100% and 0% methylation, respectively. The PCR primers are shown in Table S2.
(EPS)

Figure S9 Relative Dnmt3 mRNA expression in GVOs and Dnmt1-KO. Three biological replicates of 50 GVOs from 25 day-old C57BL/6 or [*Dnmt1^2lox/2lox^, Zp3-Cre*] females were used. All Dnmt3 mRNAs were up-regulated in Dnmt1-KO. Note that the values show relative expression levels and the actual Dnmt3b mRNA level was extremely low compared to the others. RNA extraction, quantitative reverse transcription PCR, and data analysis were carried out as described previously [26]. Briefly, RNA extraction was performed using Trizol reagent (Invitrogen) supplemented with 6.25 pg of rabbit α-globin mRNA (Sigma). After DNA digestion and reverse transcription, qPCR was performed using primer sets described previously [26]. Relative expression values were normalized to the level of rabbit α-globin mRNA. GVO was used as calibrator sample. The results are the average of the three replicates (s.d.).
(EPS)

Table S1 CG and non-CG methylation profiles in 15 ICRs.
(PDF)

Table S2 Primers for bisulfite sequencing (bisulfite-PCR).
(PDF)
