# Supplementary material for: Mouse Oocyte Methylomes at Base Resolution Reveal Genome-Wide Accumulation of Non-CpG Methylation and Role of DNA Methyltransferases
Source: PLoS Genet. 2013 Apr 18;9(4):e1003439. doi: 10.1371/journal.pgen.1003439 (PMC3630097; doi:10.1371/journal.pgen.1003439)
Supplement: Table S1 — CG and non-CG methylation profiles in 15 ICRs. (PDF) [file pgen.1003439.s010.pdf]

Table S1. CG and non-CG methylation profiles in 15 ICRs

ICR	Chr.	Start*	End	GVO		Dnmt3a-KO		Dnmt3L-KO		NGO	
				CG methylation (%)	Non-CG methylation (%)	CG methylation (%)	Non-CG methylation (%)	CG methylation (%)	Non-CG methylation (%)	CG methylation (%)	Non-CG methylation (%)
Maternally methylated ICRs											
Nespas-Gnasxl	2	174,119,863	174,126,564	95.8	5.8	9.0	0.8	6.3	0.6	3.1	1.0
Gnas1A	2	174,150,877	174,154,638	91.7	4.5	5.5	1.0	0.5	0.6	0.7	0.7
Peg10	6	4,696,743	4,699,483	93.8	4.9	22.9	0.2	20.7	0.7	19.3	0.4
Mest	6	30,684,932	30,689,966	93.4	3.8	17.4	0.8	11.5	0.3	9.2	0.9
Peg3	7	6,679,787	6,684,257	96.6	6.5	19.2	1.1	18.3	0.4	5.5	0.7
Snrpn	7	67,147,381	67,151,583	95.9	7.5	21.5	0.8	12.1	0.2	9.6	0.5
Kcnq1ot1	7	150,480,736	150,482,810	97.8	8.1	4.6	1.1	4.3	0.3	2.6	0.5
Zac1	10	12,809,697	12,812,131	97.0	4.7	6.0	0.6	3.3	0.4	0.6	0.5
Grb10	11	11,925,127	11,927,100	95.7	7.7	11.9	0.6	9.4	0.3	4.5	0.7
U2af1-rs1	11	22,871,610	22,874,212	90.0	7.2	15.5	1.4	6.4	0.3	4.8	0.6
Igf2r	17	12,934,169	12,935,816	97.4	7.6	11.9	1.1	1.4	0.6	0.8	0.4
Impact	18	13,130,435	13,133,510	97.1	6.1	7.8	0.6	14.0	0.3	16.5	0.3
Paternally methylated ICRs											
H19	7	149,764,673	149,771,930	1.0	0.5	2.4	0.4	1.5	0.3	1.1	0.6
Rasgrf1	9	89,767,090	89,775,128	4.7	0.6	1.7	0.4	1.4	0.3	1.5	0.4
Dlk1-Gtl2	12	110,762,703	110,773,093	4.4	0.4	5.2	0.4	5.5	0.5	0.9	0.6

* The genomic coordinates (mm9) of the 15 ICRs were extracted from our previous report. (Tomizawa *et al*., 2011)
