# Supplementary material for: Mouse Oocyte Methylomes at Base Resolution Reveal Genome-Wide Accumulation of Non-CpG Methylation and Role of DNA Methyltransferases
Source: PLoS Genet. 2013 Apr 18;9(4):e1003439. doi: 10.1371/journal.pgen.1003439 (PMC3630097; doi:10.1371/journal.pgen.1003439)
Supplement: Table S2 — Primers for bisulfite sequencing (bisulfite-PCR). (PDF) [file pgen.1003439.s011.pdf]

Table S2. Primers for bisulfite sequencing (bisulfite-PCR)

	Forward primer	Reverse primer
Atp8b1 top	5'-AAGTTAGTTGTTGTATTAGAAGATTATTAT-3'	5'-CTAACCTTATCTTTACATTAAAAAAAA-3'
Atp8b1 bottom	5'-TTGGTTTTGTTTTTGTATTGAAGAG-3'	5'-ACATCAAAAAATTATCACAAAAATC-3'
Igf1r top	5'-TGATTGGATATTTGATAAGATTATTTTGA-3'	5'-AAAAAATACAACATCCAACCAAAAA-3'
Igf1r bottom	5'-ATTTTGGTGATTGAAAAGTTTATTG-3'	5'-CCAATTCATCATACCTAACTATATCTT-3'
Igf2r	5'-GGGGAATTGAGGTAAGTTAGGGTTTT-3'	5'-TCTTATAACCCAAAAATCTTCACCCTAAC-3'
H19	5'-GTTTGAGGAGTTTTAAGGTAGAAGGGGATT-3'	5'-TAACAAACTCATAAATCACTCAAACATAACATTC-3'
